# Preliminary findings on diagnostic validity of the ICD-11 personality disorder scales among Croatian psychiatric patients

**DOI:** 10.1192/j.eurpsy.2023.791

**Published:** 2023-07-19

**Authors:** N. Jaksic, I. Simunovic Filipcic, M. Sagud, B. Vuksan Cusa, M. Skocic Hanzek, Z. Madzarac, V. Bilic, J. Grubisin, I. Filipcic, M. Copo, Z. Vuksan Cusa, E. Podolski, D. Marcinko

**Affiliations:** 1 Department of Psychiatry and Psychological Medicine, University Hospital Center Zagreb, Zagreb; 2 Faculty of Dental Medicine and Health, Josip Juraj Strossmayer University of Osijek, Osijek; 3 University of Zagreb School of Medicine; 4 University Psychiatric Clinic Sveti Ivan, Zagreb; 5 Neuropsychiatric Hospital “Dr. Ivan Barbot”, Popovaca, Croatia

## Abstract

**Introduction:**

Official classification of personality disorders (PDs) has witnessed a significant shift from traditional categorical to more dimensional diagnostic perspective in the recently published *International Classification of Diseases - 11th* Revision (ICD-11).

**Objectives:**

The goal of this preliminary study was to explore the clinical use (i.e., diagnostic validity) of the newly constructed, as well as recently adapted to Croatian language, self-report measures of the ICD-11 PD severity and personality trait domains.

**Methods:**

This study was carried out in a sample of 363 psychiatric patients (54% female, mean age 46 years) who were being treated at Department of Psychiatry and Psychological Medicine of the University Hospital Center Zagreb and the University Psychiatric Clinic Sveti Ivan, Zagreb, Croatia. Patients were divided into two groups: 98 patients with the diagnosis of PD (‘’PD group”) based on the ICD-10 criteria and 265 patients with other psychiatric disorders (‘’non-PD group”) (43% anxiety disorders, 31% affective disorders, 16% psychotic disorders, and 10% substance use disorders). They filled out the following scales that were developed according to the ICD-11 PD guidelines: the Personality Disorder Severity ICD-11 (PDS-ICD-11) scale and the Personality Assessment Questionnaire for ICD-11 (PAQ-11).

**Results:**

Statistical comparison of the two clinical groups revealed that the ‘’PD group” was significantly younger, had relatively more female patients and showed a trend towards higher number of psychiatric hospitalizations, whereas there were no group difference in terms of education level, relationship status and treatment length. More importantly, the ‘’PD group” exhibited a significantly higher total score (mean 16.30 vs. 12.39) on the PDS-ICD-11, indicating greater global personality dysfunction (i.e., more severe PD). Also, of the five PD trait domains measured by the PAQ-11, the ‘’PD group” had significantly more pronounced Negative Affectivity, Dissociality, Disinhibition and Detachment, whereas Anankastia was similar across the two groups.

**Conclusions:**

Given the radical changes in ICD-11 related to PD diagnosis, there is an urgent need to culturally adapt and evaluate psychometric properties of the scales developed to measure PD severity and personality trait domains. Our preliminary findings support diagnostic validity of two such instruments among Croatian psychiatric patients: the PDS-ICD-11 and PAQ-11.

**Disclosure of Interest:**

None Declared

